# Latent class analysis of the Epidemic‐Pandemic Impacts Inventory on mental health outcomes in Siyan Clinical patients

**DOI:** 10.1002/hsr2.1215

**Published:** 2023-04-20

**Authors:** Anish Shah, Olivia Arstein‐Kerslake, Michele Darling, Tiffany Morgan, Aubree Vance Torea, Helen Laines, Bhargav Joshi, Karina Pena, James Young

**Affiliations:** ^1^ Siyan Clinical Corporation Santa Rosa California USA; ^2^ LPC Consulting Associates Sacramento California USA

**Keywords:** coronavirus, COVID‐19, mental health, pandemic, social isolation, wellness

## Abstract

**Background and Aims:**

The COVID‐19 pandemic has made an outsized negative impact on mental health worldwide. However, research indicates that this impact was not uniform. This study aimed to determine how mental health patients experienced the COVID‐19 pandemic to characterize mental health disparities and identify underlying factors.

**Methods:**

We used the Epidemic‐Pandemic Impacts Inventory (EPII) and latent class analysis to determine the impacts of epidemics and pandemics across several life domains in 245 survey respondents, all of whom were mental health patients at Siyan Clinical. Respondents were predominately White (84.5%) and female (76.3%), with the majority being diagnosed with anxiety or mood disorders (76.3%).

**Results:**

In the work life domain, respondents in the higher‐impact class were more likely to be employed and/or working in healthcare. In both the home life and emotional/physical health and infection domain, respondents with mood disorders, substance use disorders, or children under 18 living at home were more likely to be in the higher‐impact class. In the home life and positive change domains, respondents that were married were more likely to be in the higher‐impact class, indicating that this group experiences more impacts from the pandemic, both positive and negative. Finally, some groups stood out as having fewer impacts from the pandemic: respondents that were male, over age 55, White, and/or have anxiety disorders were more likely to experience fewer impacts from the pandemic in the work life and home life domains.

**Conclusions:**

This study provides evidence that certain groups may experience greater or fewer impacts from the pandemic.

## 1 INTRODUCTION

The COVID‐19 pandemic has affected all our lives, particularly our mental health.^1^ However, some subgroups or populations may be more at risk for negative mental health outcomes compared to others. It is important to recognize these at‐risk groups so that they can be targeted by mental health interventional services. The COVID‐19 pandemic has provided researchers and physicians with a unique opportunity to identify the groups most at‐risk during times of extreme stress and heightened anxiety for adverse mental health outcomes.

In a recent study of mental health patients at Siyan Clinical, we used the Epidemic‐Pandemic Impacts Inventory (EPII) survey recently developed by Grasso and colleagues^2^, ^3^ and found that the age groups least affected by COVID‐19 included individuals aged 65 years and older.^4^ This observation is in line with other research that has also noted older age may buffer against the negative impact of the COVID‐19 pandemic on mental health.^5^ Similarly, another study found that younger people self‐reported more negative mental health outcomes during the COVID‐19 pandemic.^4^, ^6^ Perceived social support may also buffer against the negative effects of the COVID‐19 pandemic.^6^, ^7^ We also observed that people with children under the age of 18 years also reported more positive indicators associated with the pandemic compared to those without children at home, although it is worth noting that this same group also noted more negative indicators on the EPII survey.^4^

We further found that genderqueer, nonconforming, and transgender individuals may also be at higher risk for more negative impacts associated with the pandemic.^4^ This observation, too, is in line with other recent research focused on transgender individuals, as it was reported that transgender individuals in India were at increased emotional and social risks during the pandemic.^8^ Without a doubt, more inclusive research is needed to understand the unique challenges populations such as these face.^9^

In addition to these aforementioned groups (younger individuals, people with limited social support, and transgender/gender nonconforming individuals), it is likely that there are other subpopulations that are at an increased risk for negative effects associated with large‐scale stressors such as the COVID‐19 pandemic. Here, we sought to understand our prior study further by doing multivariate analyses on this data set with the goal of identifying those most at‐risk during the ongoing pandemic.

## 2 MATERIALS AND METHODS

### 2.1 Participants

As described previously,^4^ approximately 3500 adult patients in the Siyan Clinical Corporation and Siyan Clinical Research practices were invited to participate in a one‐time anonymous survey to assess the impacts of the COVID‐19 pandemic across multiple life domains. Interested patients signed and returned an informed consent form and then received a link to the online survey. A total of 326 patients completed the consent form and received the survey link from Siyan. Of these 326 patients, a total of 245 people responded from November 9, 2020, to February 18, 2021, for a completion rate of 75.2% among those who had completed the consent form. Patients received a US $10 eGift card from Starbucks upon completion of the survey. Participants were eligible for inclusion if they received or were receiving care from Siyan Clinical, were between the ages of 18 and 80, and could understand and voluntarily agree to an informed e‐consent form. Patients outside of the age range and those with a known diagnosis of dementia were not included.

These procedures were reviewed by the Advarra Institutional Review Board (IRB) and written informed consent was obtained from all participants. A notice of intent was sent to the authors of the EPII survey before IRB submission. In September 2020, the protocol was determined to be IRB exempt by Advarra. This study was conducted using ethical principles derived from international guidelines including the Declaration of Helsinki and the Council for International Organizations of Medical Sciences International Ethical Guidelines. This study was reviewed and published on ClinicalTrials.gov (NCT04568135) on September 29, 2020.

### 2.2 The EPII

We used the EPII tool as described previously.^4^ The EPII is a newly developed, 92‐item tool designed to determine the impacts of epidemics and pandemics in personal and social life domains developed by Grasso and colleagues.^3^, ^10^ Briefly, the EPII is divided into 10 subcategories, with a different number of indicators in each subcategory: work and employment, education and training, home life, social activities, economic activities, emotional health and well‐being, physical health problems, physical distancing and quarantine, infection history, and positive change. All domains except for positive change indicate negative or adverse experiences. Respondents were presented with indicators in each subcategory and asked questions such as, “Since the coronavirus disease pandemic began, what has changed for you or your family?” Participants then respond with yes (me), yes (person in home), no, or N/A (not applicable).

### 2.3 Data and statistical analysis

Latent class analysis (LCA) is a statistical method to identify differences between subgroups that share certain characteristics. LCA identifies subgroups (latent classes) by an observed response pattern to categorical indicator variables. Analysis was conducted using the poLCA package for R.^11^ This statistical package uses expectation maximization and Newton–Raphson algorithms to obtain maximum likelihood estimates of model parameters. EPII items with an endorsement rate of less than 5% were excluded from LCAs, with 12 of the 92 questions (13.0%) omitted. Following a previous 2020 analysis by Grasso et al.,^2^ EPII domains were combined into broader conceptual categories: work life (11 indicators), home life (15 indicators), social activities and isolation (17 indicators), emotional/physical health and infection (18 indicators), and positive change (19 indicators). LCA was then applied separately to these broader conceptual categories. Item endorsement rates are presented in Supporting Information: Table 1.

For each domain, LCA models with the number of classes ranging from one to six were created. The final number of latent classes for each domain was identified by evaluating the Bayesian Information Criterion (BIC), with a lower BIC indicating better model fit.^12^ Entropy was also examined as a diagnostic criterion, with values above 0.8 more desirable, although no definitive cutoff criterion exists.^13^ After determining the best‐fitting class solution for each domain, predicted class membership was compared to a set of dichotomous demographic characteristics using *χ* ^2^ test to identify differences between classes. A *p* < 0.05 was considered statistically significant. For domains with a best‐fitting class solution of greater than two, post hoc *χ* ^2^ tests following the initial *χ* ^2^ were conducted with a Bonferroni adjustment (*p* < 0.02).

## 3 RESULTS

### 3.1 Sample characteristics

Table 1 presents the sociodemographic characteristics of the sample. Most of the sample self‐identified as female (76.3%) and White (84.5%). About half (54.2%) of the sample reported being currently employed. The majority of the sample reported being diagnosed with mood disorders (76.3%) or anxiety disorders (76.3%). Almost half (47.8%) of the sample reported earning a bachelor's degree or higher.

**Table 1:** Sociodemographic characteristics of Siyan Clinical patients responding to the survey.

Sociodemographic characteristics		
Variable	*n*	%
Age		
18–24	25	10.2
25–34	39	15.9
35–44	57	23.3
45–54	40	16.3
55–64	47	19.2
65–74	24	9.8
75 or older	13	5.3
Gender		
Female	187	76.3
Male	52	21.2
Genderqueer/gender nonconforming	3	1.2
Transgender male	2	0.8
Other	1	0.4
Ethnicity		
American Indian/Alaska Native	7	2.1
Asian	5	2
Black/African American	5	2.0
Hispanic/Latino	24	9.8
White	207	84.5
Other	6	2.4
Nondisclosed	9	3.7
Education		
Less than high school	3	1.2
Graduated high school	20	8.2
Trade/technical school	8	3.3
Some college, no degree	62	25.3
Associate degree	34	13.9
Bachelor's degree	74	30.2
Advanced degree (Master's, PhD, MD)	43	17.6
Nondisclosed	1	0.4
Children under 18 living at home		
Yes	80	32.7
No	162	66.1
Nondisclosed	3	1.2
Employment status		
Employed, full time	98	40.0
Employed, part time	35	14.3
Unemployed, disabled	22	9.0
Unemployed, looking for work	19	7.8
Unemployed, not looking for work	6	2.4
Unemployed, retired	32	13.1
Unemployed, volunteer work	1	0.4
Other—write In	29	11.8
Nondisclosed	3	1.2
Mental health diagnosis		
Anxiety disorders	187	76.3
Eating disorders	50	20.4
Mood disorders	187	76.3
Personality disorders	8	3.3
Psychotic disorders	2	0.8
Substance abuse disorders	29	11.8
Trauma‐related disorders	75	30.6
Other	19	7.8
Nondisclosed	7	2.9
Marital status		
Divorced	33	13.5
Married/domestic partner	135	55.1
Separated	7	2.9
Single/never married	58	23.7
Widowed	9	3.7
Other	1	0.4
Nondisclosed	1	0.4

### 3.2 Summary of analysis

All fit statistics are presented in Supporting Information: Tables 2–6 and the best‐modeled class is highlighted in bold. Work life, emotional/physical health and infection, and positive change domains had best‐fitting class solutions of 2, whereas home life and social activities/isolation had best‐fitting solutions of three classes. Table 2 presents the average posterior probabilities for class membership in the EPII domains. The average posterior probabilities for most likely class membership were high, ranging from 0.87 to 0.99. The LCAs and demographic class differences by domain are presented below.

**Table 2:** Average posterior probabilities for latent class analysis of each EPII domain.

Average posterior probabilities for LCAs			
	Most likely latent class membership		
Classification	1	2	3
Work life			
Class 1	0.97	0.03	–
Class 2	0.05	0.95	–
Home life			
Class 1	0.99	0	0
Class 2	0.01	0.92	0.07
Class 3	0.04	0.05	0.91
Social/isolation			
Class 1	0.87	0.07	0.06
Class 2	0.07	0.87	0.06
Class 3	0.06	0.03	0.91
Emotional/physical			
Class 1	0.94	0.06	–
Class 2	0.09	0.91	–
Positive change			
Class 1	0.91	0.09	–
Class 2	0.05	0.95	–

Abbreviations: EPII, Epidemic‐Pandemic Impacts Inventory; LCA, latent class analysis.

### 3.3 Work

The work life LCA had a best‐fitting model of two classes with 11 indicators (Supporting Information: Table 2). The two classes were split nearly evenly: Class 1 comprised 45.9% of the sample and Class 2 comprised 54.1% of the sample (see Table 3).

**Table 3:** Class distribution of respondents by EPII domain.

Class distribution by domain						
	Class 1 (*n*)	Class 1 (%)	Class 2 (*n*)	Class 2 (%)	Class 3 (*n*)	Class 3 (%)
Work life	113	45.9	132	54.1	–	–
Home life	69	28.2	130	53.1	46.55	18.7
Social/isolation	86	35.1	64	26.4	93.1	38.4
Emotional/physical	140	56.5	108	43.5	–	–
Positive	100	41.4	145	58.6	–	–

Abbreviation: EPII, Epidemic‐Pandemic Impacts Inventory.

A visualization of the class differentiation is illustrated in Supporting Information: Figure 1. Class 1 had a higher conditional probability of item endorsements on average for all work life domain items compared to Class 2 (Class 1: 0.49, Class 2: 0.14). Items with the largest differentiation between classes included:

- Had to continue to work even though in close contact with people who might be infected (Class 1: 0.88, Class 2: 0.12).
- Spend a lot of time disinfecting at home due to close contact with people who might be infected at work (Class 1: 0.79, Class 2: 0.06).
- Increase in workload or work responsibilities (Class 1: 0.85, Class 2: 0.20).

When comparing the classes on this domain, several interesting differences were noted (Table 1). Class 1 had a greater proportion of respondents than Class 2 that reported being employed and working in a healthcare setting. Class 2 had a greater proportion of respondents than Class 1 that were over age 55 and identified as White.

Overall, respondents experiencing greater impact (Class 1) of the pandemic in the work life domain were more likely to be employed and/or more likely to be working in healthcare, while respondents experiencing less impact (Class 2) in their work life were over age 55 and/or White.

### 3.4 Home life

The home life LCA had a best‐fitting model of three classes with 15 indicators (see Supporting Information: Table 3). For home life, the sample was split into three classes. About half (53.1%) of participants were grouped into Class 2, followed by 28.2% in Class 1, and 18.7% in Class 3 (see Table 3).

Class differentiation is visualized in Supporting Information: Figure 2. Class 1 had the highest average rates of endorsement on items compared to Classes 2 and 3, with Class 2 being extremely low compared to the other classes (Class 1: 0.44, Class 2: 0.06, Class 3: 0.26). Items with the largest differentiation between classes included:

- Childcare or babysitting unavailable when needed (Class 1: 0.57, Class 2: 0.01, Class 3: 0.00).
- Difficulty taking care of children in the home (Class 1: 0.80, Class 2: 0.00, Class 3: 0.00).
- Had to take over teaching or instructing a child (Class 1: 0.78, Class 2: 0.00, Class 3: 0.00).
- More conflict with child or harsher in disciplining child or children (Class 1: 0.74, Class 2: 0.00, Class 3: 0.14).

- Had a child in home who could not go to school (Class 1: 0.97, Class 2: 0.14, Class 3: 0.15).
- Increase in verbal arguments or conflict with a partner or spouse (Class 1: 0.63, Class 2: 0.16, Class 3: 0.66).

When comparing the three classes on the home domain, several differences were noted (see Table 3). Class 1 had a greater proportion of respondents than Class 2 and 3 with children under 18 living at home and a greater proportion of respondents who are married. Class 2 had a greater proportion of respondents than Classes 1 and 3 over age 55. Class 3 had a greater proportion of respondents than Class 2 with a trauma disorder. Class 3 also had a greater proportion of respondents than Classes 1 and 2 under age 34. Further, Class 3 had a greater proportion of respondents than Class 1 with an eating disorder. Finally, Class 1 had a lower proportion of respondents identifying as White compared to Class 2.

Overall, respondents experiencing greater impacts (Class 1) in their home life were more likely to be married and/or have children under 18 living in the home. Respondents with less impacts (Class 2) were more likely to be over age 55 and/or White. Respondents with moderate impacts (Class 3) were more likely to have reported a trauma disorder, eating disorder, and/or be under age 34.

### 3.5 Social activities and isolation

The social activities and isolation LCA had a best‐fitting model of three classes with 17 indicators (see Supporting Information: Table 4). Like home life, social activities, and isolation could be broken into three distinct classes. Class 3 had the largest percentage of respondents (38.4%), followed by Class 1 (35.1%) and Class 2 (26.4%; see Table 1).

Class differentiation is visualized in Supporting Information: Figure 3. Class 3 had the highest average endorsement rate across items, followed by Classes 1 and 2 (Class 1: 0.47, Class 2: 0.31, Class 3: 0.63). Items with the largest differentiation between classes included:

- Isolated or quarantined due to possible exposure of the disease (Class 1: 0.20, Class 2: 0.41, Class 3: 0.96).
- Unable to participate in social clubs, sports team, or usual volunteer activities (Class 1: 0.95, Class 2: 0.41, Class 3: 0.57).
- Isolated or quarantined due to symptoms of this disease (Class 1: 0, Class 2: 0.16, Class 3: 0.57).

When comparing the classes on social activities and isolation (Supporting Information: Table 4), we noted that Class 2 had a greater proportion of respondents than Classes 1 and 3 that identified as male, while Classes 2 and 3 had a greater proportion of respondents than Class 1 reporting substance abuse.

Overall, class differences were limited in the social activities and isolation domain. This may be due to generally high rates of item endorsement in this domain (see Supporting Information: Table 1), reflected also in the high average conditional probabilities of item endorsement (Class 1: 0.47, Class 2: 0.31, Class 3: 0.63). Additionally, the California statewide shelter‐in‐place order, which mandated social distancing, was implemented on December 3, 2020, and lifted on January 25, 2020, which means that many survey respondents would have completed the survey after experiencing the shelter‐in‐place order. Items in this domain are related to activities that were limited or prohibited by the shelter‐in‐place order and may account for the high rates of item endorsement.

In short, respondents experiencing less impact (Class 2) in the social activities and isolation domain were more likely to identify as male. Respondents experiencing moderate impacts (Class 1) were more likely to not report substance abuse compared to people reporting substance abuse, who were more likely to experience less impact (Class 2) or greater impact (Class 3).

### 3.6 Emotional/physical health and infection

The emotional/physical health and infection LCA had a best‐fitting model of two classes with 18 indicators (see Supporting Information: Table 5). The two classes were mostly evenly split, with 56% of participants in Class 1 and 43.5% of participants in Class 2 (see Table 3).

Class differentiation is visualized in Supporting Information: Figure 4. Class 2 had a higher average endorsement probability compared to Class 1 (Class 1: 0.35, Class 2: 0.59). Items with the largest differentiation between classes included:

- Received less medical care than usual (Class 1: 0.33, Class 2: 0.86).
- Increase in health problems not related to this issue (Class 1: 0.27, Class 2: 0.80).
- Increase in sleep problems or poor sleep quality (Class 1: 0.62, Class 2: 1).

When making class comparisons (Table 3), Class 2 (the class that experienced greater impacts) had a greater proportion of respondents than Class 1 with children under 18 living at home, report a trauma disorder, and/or report a mood disorder.

### 3.7 Positive change

The positive change domain had a best‐fitting model of two classes with 19 indicators (see Supporting Information: Table 6). The classes were mostly evenly split, with 58.6% of participants in Class 2 and 41.4% of participants in Class 1 (see Table 3).

Class differentiation is visualized in Supporting Information: Figure 5. Class 1 had a higher average probability of item endorsement compared to Class 2 for the positive change domain (Class 1: 0.53, Class 2: 0.23). Items with the largest differentiation between classes included:

- Paid more attention to personal health (Class 1: 0.91, Class 2: 0.34).
- Paid more attention to preventing physical injuries (Class 1: 0.64, Class 2: 0.26).
- Ate healthier foods (Class 1: 0.63, Class 2: 0.15).
- Improved relationships with family or friends (Class 1: 0.68, Class 2: 0.20).

Overall, a systematic comparison of the classes (Table 3) revealed that Class 1 (who experienced greater positive impacts according to the EPII survey) had a greater proportion of respondents than Class 2 who are married and/or report an anxiety disorder.

## 4 DISCUSSION

Overall, we show here that certain groups may experience greater or fewer impacts from the pandemic depending on a given aspect of their lives. We found that employment (particularly employment in healthcare) was associated with higher impact from the pandemic. Having children under the age of 18 was also associated with a higher impact. Being married was associated with both high negative impact and high positive change, interestingly. Males were less likely to be impacted on the social activities and isolation domain. Additionally, older individuals (aged 55 years and older) and White individuals were also less likely to report higher impacts from the COVID‐19 pandemic. Altogether, this multivariate analysis adds greatly to our previously reported findings from this data set. These data also complement the growing body of literature on the impacts of COVID‐19.

The results for our study comport with the growing consensus within the literature that identifies disparities in the effects of the COVID‐19 pandemic on the mental health of specific groups. For example, our findings that older people were less impacted by the pandemic compared to their younger peers is in line with several reports showing that adolescents were particularly impacted by the pandemic.^14^, ^15^ Two studies of Italian teenage students found that students experienced significant sadness throughout the pandemic, with many citing the lack of an in‐person community as a reason.^14^, ^15^ Interestingly, these studies and others found that male students experienced fewer symptoms than their female counterparts, a phenomenon highlighted in the current work.^14^, ^15^, ^16^ Such gender differences were mirrored in our results, wherein male respondents were more likely to be sorted into Class 2 and experienced less severe effects on mental health from the pandemic. These results are in line with other reports finding that women bore the brunt of the pandemic in many cases due to increased caregiver activities and household chores, both of which were also observed in the current study.^16^, ^17^, ^18^ Other studies indicate that gender‐related disparities in mental health during the pandemic are also tied to domestic violence and unaccompanied birth, warranting future research.^19^, ^20^ Despite the relationship with marriage and domestic abuse during the pandemic, marriage is considered a protective factor in terms of mental health.^21^

Another critical set of factors that influence COVID‐19 pandemic‐related mental health impacts is pre‐existing mental illness. Those living with mood disorders tended to suffer a greater impact from the pandemic, a result that mirrors previous research.^22^ However, our data suggest that people living with anxiety before the pandemic were less affected. This finding necessitates further research, as other reports have noted a spike in anxiety due to the pandemic.^23^, ^24^ In addition, physical illness plays a role in the degree of impact an individual faced during the pandemic, particularly COVID‐19 itself.^25^ Both hospitalized and nonhospitalized patients display increased symptoms of posttraumatic stress disorder (PTSD), anxiety, and depression. However, increased symptoms were noted in people who were hospitalized due to the illness.^25^

Understanding who is most at risk for impact by stressors such as pandemics is important because early intervention is key. Moreover, being aware of how to cope with an event such as the COVID‐19 pandemic can help individuals with their mental health. For instance, one study found that coping strategies are significant predictors for mental health measures; therefore, education about positive thinking, active coping, and social support could be beneficial for dealing with a decrease in mental health.^26^ Poor mental health is often associated with poor coping mechanisms, as was recently demonstrated by Eastman et al. when they observed that mental health symptomatology was associated with increased drinking since the COVID‐19 pandemic onset.^27^ This particularly affected younger people, wherein binge drinking and vaping rates have decreased among adolescents while cannabis use and total alcohol use have gone up.^28^ Given that we previously observed an increase in substance use,^4^ and substance abuse surfaced as related to high impact here as well, this is a particularly important negative coping method that should be a topic of both education and intervention among vulnerable groups.

This study is not without limitations. The main limitation for this analysis is the sample size. While there is no exact rule on the number of cases necessary for LCA, studies by Nylund‐Gibson and Choi suggest that 300 or more cases is desirable.^29^ However, smaller samples may suffice with simpler models (fewer indicators and classes). The present analysis includes 245 cases, limiting the power of this study. These data also solely included adult (over the age of 18 years) patients of Siyan Clinical who responded to our survey. The scientific literature would benefit immensely from further research aimed at youth. Children and adolescents also experience mental health concerns, and knowing which factors make them most vulnerable would be immensely beneficial. For instance, research has already shown that COVID‐19 is detrimental for the mental health of children and adolescents and causes high rates of anxiety, depression, and posttraumatic symptoms.^30^ Further, LGBTQ+ youth have also experienced unique stressors during the pandemic.^31^ In addition to these concerns, the psychometric quality of the EPII is yet to be validated and an optimal range has not been assigned. These features may limit its use in diverse populations. This study is also limited in that there were so few transgender/genderqueer/gender nonconforming individuals, thus making their data difficult to parse apart in these analyses. However, these vulnerable populations have previously been shown to be greatly impacted by the COVID‐19 pandemic,^9^, ^32^ and future research should be aimed at determining what other risk factors these groups have. Additionally, we did not measure sexual orientation in this survey, but other research has indeed shown that the LGBTQ+ community exhibited high levels of depression, stress, and experienced discrimination during the COVID‐19 pandemic.^33^ Finally, participants were not sorted by previous or current COVID‐19 infection status, which is known to have substantial negative lingering impacts on mental health, heightening symptoms of depression and PTSD.^25^, ^34^ Future work should focus on individuals who have recovered, or are recovering from, the illness.

## 5 CONCLUSIONS

In conclusion, this study provides evidence that certain groups may experience greater or fewer impacts from the pandemic depending on a given aspect of their lives. In the work life domain, respondents in the higher‐impact class were more likely to be employed and/or working in healthcare. In both the home life and emotional/physical health and infection domain, respondents with children under 18 living at home were more likely to be in the higher‐impact class. Interestingly, in the home life and positive change domains, respondents that were married were more likely to be in the higher‐impact class, indicating that this group experiences more impacts from the pandemic, both positive and negative. Finally, a few groups stood out as having fewer impacts from the pandemic: respondents who were over age 55 and/or White were more likely to experience fewer impacts from the pandemic in the work life and home life domains.

## AUTHOR CONTRIBUTIONS

**Anish Shah**: Conceptualization; data curation; funding acquisition; investigation; methodology; project administration; resources; supervision; writing—original draft. **Olivia Arstein‐Kerslake**: Data curation; formal analysis; investigation; methodology; resources; software; visualization; writing—original draft. **Michele Darling**: Data curation; formal analysis; investigation; methodology; resources; software; validation; visualization; writing—original draft; writing—review and editing. **Tiffany Morgan**: Conceptualization; data curation; formal analysis; investigation; software; supervision; validation; visualization; writing—original draft; writing—review and editing. **Aubree Vance Torea**: Conceptualization; data curation; formal analysis; investigation; software; visualization; writing—original draft; writing—review and editing. **Helen Laines**: Conceptualization; data curation; formal analysis; methodology; software; visualization; writing—original draft; writing—review and editing. **Bhargav Joshi**: Conceptualization; data curation; formal analysis; resources; validation; visualization; writing—original draft. **Karina Pena**: Data curation; formal analysis; investigation; methodology; software; validation; visualization; writing—original draft; writing—review and editing. **James Young**: Conceptualization; data curation; formal analysis; investigation; methodology; resources; writing—original draft; writing—review and editing.

## CONFLICT OF INTEREST STATEMENT

The authors declare no conflict of interest.

## ETHICS STATEMENT

These procedures were reviewed by the Advarra IRB and conducted using ethical principles derived from international guidelines including the Declaration of Helsinki and the Council for International Organizations of Medical Sciences International Ethical Guidelines. Informed consent was obtained from all subjects involved in the study.

## TRANSPARENCY STATEMENT

The lead author Anish Shah affirms that this manuscript is an honest, accurate, and transparent account of the study being reported; that no important aspects of the study have been omitted; and that any discrepancies from the study as planned (and, if relevant, registered) have been explained.

## Supporting information

Supporting information.

Supplemental Figure 1.

Supplemental Figure 2.

Supplemental Figure 3.

Supplemental Figure 4.

Supplemental Figure 5.

## Data Availability

Data will be made available upon request.
