# Supplementary material for: Latent class analysis of the Epidemic‐Pandemic Impacts Inventory on mental health outcomes in Siyan Clinical patients
Source: Health Sci Rep. 2023 Apr 20;6(4):e1215. doi: 10.1002/hsr2.1215 (PMC10119487; doi:10.1002/hsr2.1215)
Supplement: Supplementary file 1 — Supporting information. [file HSR2-6-e1215-s003.docx]

# Latent Class Analysis of The Epidemic-Pandemic Impacts Inventory on Mental Health Outcomes in XXX Clinical Patients

# Supplemental Results

**Supplemental Table 1.**

| **Item Endorsement Rates (“Yes, me” or “Yes, person in home”)** |  |  |
| --- | --- | --- |
|  | Count  (n=245) | Percent  (%) |
| **Work Life** |  |  |
| Increase in workload or work responsibilities. | 121 | 49.8 |
| Had to continue to work even though in close contact with people who might be infected (e.g., customers, patients, co-workers) | 113 | 46.5 |
| Reduced work hours or furloughed | 110 | 44.9 |
| Spend a lot of time disinfecting at home due to close contact with people who might be infected at work. | 95 | 38.9 |
| Hard time making the transition to working from home. | 78 | 32.2 |
| Laid off from job or had to close own business | 78 | 32.1 |
| Hard time doing job well because of needing to take care of people in the home | 70 | 28.8 |
| Had to lay-off or furlough employees or people supervised | 29 | 12.0 |
| Provided direct care to people with the disease (e.g., doctor, nurse, patient care assistant, radiologist). | 24 | 9.9 |
| Provided supportive care to people with the disease (e.g., medical support staff, custodial, administration). | 24 | 9.9 |
| Provided care to people who died as a result of the disease. * | 8 | 3.3 |
| **Home Life** |  |  |
| Increase in verbal arguments or conflict with a partner or spouse. | 94 | 38.5 |
| Had a child in home who could not go to school. | 91 | 37.3 |
| Had to spend a lot more time taking care of a family member. | 68 | 27.9 |
| Increase in verbal arguments or conflict with other adult(s) in home. | 67 | 27.5 |
| Had to take over teaching or instructing a child. | 63 | 25.8 |
| More conflict with child or harsher in disciplining child or children. | 57 | 23.4 |
| Difficulty taking care of children in the home | 56 | 23.0 |
| Unable to pay important bills like rent or utilities. | 43 | 17.6 |
| Childcare or babysitting unavailable when needed. | 41 | 16.8 |
| Had to move or relocate. | 33 | 13.6 |
| Difficulty getting places due to less access to public transportation or concerns about safety. | 33 | 13.5 |
| Family or friends had to move into your home. | 32 | 13.2 |
| Unable to get needed medications (e.g., prescriptions or over-the-counter). | 26 | 10.7 |
| Unable to get enough food or healthy food. | 26 | 10.6 |
| Increase in physical conflict among children in home. | 19 | 7.8 |
| Became homeless.* | 10 | 4.1 |
| Increase in physical conflict with a partner or spouse.* | 10 | 4.1 |
| Increase in physical conflict with other adult(s) in home.* | 7 | 2.9 |
| Unable to access clean water.* | 2 | 0.8 |
| **Social Activities and Isolation** |  |  |
| Family celebrations cancelled or restricted. | 235 | 96.3 |
| Separated from family or close friends. | 218 | 89.3 |
| Planned travel or vacations cancelled. | 209 | 86.0 |
| Unable to do enjoyable activities or hobbies. | 209 | 86.0 |
| Unable to participate in social clubs, sports teams, or usual volunteer activities. | 197 | 80.4 |
| Isolated or quarantined due to possible exposure to this disease. | 134 | 54.7 |
| Limited physical closeness with child or loved one due to concerns of infection. | 130 | 53.1 |
| Religious or spiritual activities cancelled or restricted. | 118 | 48.2 |
| Isolated due to existing health conditions that increase risk of infection or disease. | 98 | 40.2 |
| Close family member not in the home was quarantined. | 84 | 34.3 |
| Unable to attend in-person funeral or religious services for a family member or friend who died. | 80 | 32.7 |
| Unable to be with a close family member in critical condition. | 71 | 29.0 |
| Isolated or quarantined due to symptoms of this disease. | 64 | 26.2 |
| Unable to visit loved one in a care facility (e.g., nursing home, group home). | 59 | 24.2 |
| Entire household was quarantined for a week or longer. | 46 | 18.8 |
| Did not have the ability or resources to talk to family or friends while separated. | 44 | 18.1 |
| Family member was unable to return home due to quarantine or travel restrictions. | 26 | 10.6 |
| Moved out or lived away from family due to high-risk job (e.g., health care worker, first responder).* | 12 | 4.9 |
| **Emotional/Physical Health and Infection** |  |  |
| Increase in use of alcohol or substances. | 228 | 93.1 |
| Important medical procedure cancelled (e.g., surgery). | 227 | 92.7 |
| Increase in child's sleep difficulties or nightmares. | 223 | 91.0 |
| Increase in health problems not related to this disease. | 206 | 84.1 |
| Increase in mental health problems or symptoms (e.g., mood, anxiety, stress). | 192 | 78.7 |
| Got less medical care than usual (e.g., routine or preventive care appointments). | 182 | 74.6 |
| Less physical activity or exercise. | 138 | 56.6 |
| Tested and currently have this disease. | 123 | 50.2 |
| Increase in sleep problems or poor sleep quality. | 86 | 35.1 |
| Not satisfied with changes in mental health treatment or therapy. | 81 | 33.1 |
| Spent more time on screens and devices (e.g., looking at phone, playing video games, watching TV). | 56 | 22.9 |
| Increase in child behavioral or emotional problems. | 55 | 22.6 |
| Unable to access mental health treatment or therapy. | 41 | 16.9 |
| Overeating or eating more unhealthy foods (e.g., junk food). | 41 | 16.7 |
| Unable to access medical care for a serious condition (e.g., dialysis, chemotherapy). | 33 | 13.8 |
| Elderly or disabled family members not in the home unable to get the help they need. | 33 | 13.8 |
| Currently have symptoms of this disease but have not been tested. | 18 | 7.4 |
| More time sitting down or being sedentary. | 13 | 5.3 |
| Got medical treatment due to severe symptoms of this disease. * | 8 | 3.3 |
| Hospital stay due to this disease. * | 7 | 2.9 |
| Someone died of this disease while in our home. * | 4 | 1.6 |
| Tested positive for this disease but no longer have it. * | 3 | 1.2 |
| Death of close friend or family member for this disease. * | 1 | 0.4 |
| Had symptoms of this disease but never tested. * | 0 | 0.0 |
| **Positive Change** |  |  |
| More appreciative of things usually taken for granted. | 192 | 78.7 |
| More quality time with family or friends in person or from a distance (e.g., on the phone, Email, social media, video conferencing, online gaming). | 141 | 57.8 |
| Paid more attention to personal health. | 141 | 57.6 |
| More quality time with partner or spouse. | 118 | 48.2 |
| More time doing enjoyable activities (e.g., reading books, puzzles). | 113 | 46.1 |
| Paid more attention to preventing physical injuries. | 103 | 42.0 |
| Improved relationships with family or friends. | 97 | 39.6 |
| More time in nature or being outdoors. | 93 | 38.0 |
| More quality time with children. | 89 | 36.6 |
| Ate healthier foods. | 85 | 34.7 |
| New connections made with supportive people. | 83 | 34.0 |
| Developed new hobbies or activities. | 82 | 33.5 |
| Donated time or goods to a cause related to this disease (e.g., made masks, donated blood, volunteered). | 66 | 26.9 |
| Found greater meaning in work, employment, or school. | 62 | 25.3 |
| Volunteered time to help people in need. | 48 | 19.6 |
| Increase in exercise or physical activity. | 44 | 18.0 |
| Less use of alcohol or substances. | 41 | 16.9 |
| More efficient or productive in work, employment, or school. | 40 | 16.5 |
| Spent less time on screens or devices outside of work hours (e.g., looking at phone, playing video games, watching TV). | 17 | 7.0 |
| *Items not included in LCA models since endorsement < 5% |  |  |

**Supplemental Table 2.**

| **Fit Statistics and Diagnostic Criteria for Work Life LCA** | | | | | | | | | | |
| --- | --- | --- | --- | --- | --- | --- | --- | --- | --- | --- |
| **Classes** | **log-likelihood** | **resid. df** | **BIC** | **aBIC** | **cAIC** | **likelihood- ratio** | **Entropy** | **Parameters** | **Smallest class count  (n)** | **Smallest class size  (%)** |
| Model 1 | -1484.59 | 234 | 3029.70 | 2994.83 | 3040.70 | 842.89 | - | 11 | 245 | 100 |
| **Model 2** | **-1325.35** | **222** | **2777.23** | **2704.32** | **2800.23** | **540.04** | **0.816** | **23** | **113** | **46** |
| Model 3 | -1294.95 | 210 | 2782.44 | 2671.49 | 2817.44 | 478.88 | 0.827 | 35 | 39 | 16 |
| Model 4 | -1278.64 | 198 | 2815.83 | 2666.85 | 2862.83 | 447.44 | 0.834 | 47 | 12 | 5 |
| Model 5 | -1260.92 | 186 | 2846.42 | 2659.39 | 2905.42 | 411.48 | 0.784 | 59 | 12 | 5 |
| Model 6 | -1246.72 | 174 | 2884.04 | 2658.97 | 2955.04 | 383.24 | 0.542 | 71 | 7 | 3 |

**Supplemental Table 3.**

| **Fit Statistics and Diagnostic Criteria for Home Life LCA** | | | | | | | | | | |
| --- | --- | --- | --- | --- | --- | --- | --- | --- | --- | --- |
| **Classes** | **log-likelihood** | **resid. df** | **BIC** | **aBIC** | **cAIC** | **likelihood-ratio** | **Entropy** | **Parameters** | **Smallest class count (n)** | **Smallest class size(%)** |
| Model 1 | -1759.54 | 230 | 3601.59 | 3554.04 | 3616.59 | 1507.71 | - | 15 | 245 | 100 |
| Model 2 | -1481.93 | 214 | 3134.40 | 3036.13 | 3165.40 | 960.75 | 0.941 | 31 | 71 | 29 |
| **Model 3** | **-1420.06** | **198** | **3098.67** | **2949.69** | **3145.67** | **841.09** | **0.848** | **47** | **47** | **19** |
| Model 4 | -1383.79 | 182 | 3114.17 | 2914.46 | 3177.17 | 767.71 | 0.858 | 63 | 10 | 4 |
| Model 5 | -1361.21 | 166 | 3157.03 | 2906.60 | 3236.03 | 722.58 | 0.777 | 79 | 10 | 4 |
| Model 6 | -1337.65 | 150 | 3197.92 | 2896.78 | 3292.92 | 674.68 | 0.855 | 95 | 10 | 4 |

**Supplemental Table 4.**

| **Fit Statistics and Diagnostic Criteria for Social Activities and Isolation LCA** | | | | | | | | | | |
| --- | --- | --- | --- | --- | --- | --- | --- | --- | --- | --- |
| **Classes** | **log-likelihood** | **resid. df** | **BIC** | **aBIC** | **cAIC** | **likelihood-ratio** | **Entropy** | **Parameters** | **Smallest class count (n)** | **Smallest class size(%)** |
| Model 1 | -2162.68 | 228 | 4418.87 | 4364.98 | 4435.87 | 1672.62 | - | 17 | 245 | 100 |
| Model 2 | -2058.94 | 210 | 4310.42 | 4199.47 | 4345.42 | 1478.15 | 0.663 | 35 | 120 | 49 |
| **Model 3** | **-2009.69** | **192** | **4310.94** | **4142.94** | **4363.94** | **1387.15** | **0.741** | **53** | **64** | **26** |
| Model 4 | -1967.88 | 174 | 4326.36 | 4101.29 | 4397.36 | 1308.87 | 0.775 | 71 | 37 | 15 |
| Model 5 | -1947.57 | 156 | 4384.74 | 4102.62 | 4473.74 | 1269.79 | 0.761 | 89 | 22 | 9 |
| Model 6 | -1929.96 | 138 | 4448.56 | 4109.38 | 4555.56 | 1235.60 | 0.758 | 107 | 15 | 6 |

**Supplemental Table 5*.***

| **Fit Statistics and Diagnostic Criteria for Emotional/Physical Health and Infection LCA** | | | | | | | | | | |
| --- | --- | --- | --- | --- | --- | --- | --- | --- | --- | --- |
| **Classes** | **log-likelihood** | **resid. df** | **BIC** | **aBIC** | **cAIC** | **likelihood-ratio** | **Entropy** | **Parameters** | **Smallest class count (n)** | **Smallest class size(%)** |
| Model 1 | -2040.31 | 227 | 4179.64 | 4122.58 | 4197.64 | 1461.64 | - | 18 | 245 | 100 |
| **Model 2** | **-1907.75** | **208** | **4019.05** | **3901.76** | **4056.05** | **1208.99** | **0.746** | **37** | **105** | **43** |
| Model 3 | -1863.22 | 189 | 4034.50 | 3856.99 | 4090.50 | 1124.91 | 0.744 | 56 | 42 | 17 |
| Model 4 | -1823.74 | 170 | 4060.08 | 3822.34 | 4135.08 | 1055.80 | 0.763 | 75 | 12 | 5 |
| Model 5 | -1798.01 | 151 | 4113.14 | 3815.17 | 4207.14 | 1006.57 | 0.735 | 94 | 10 | 4 |
| Model 6 | -1781.89 | 132 | 4185.41 | 3827.21 | 4298.41 | 976.06 | 0.795 | 113 | 15 | 6 |

**Supplemental Table 6.**

| **Fit Statistics and Diagnostic Criteria for Positive Change LCA** | | | | | | | | | | |
| --- | --- | --- | --- | --- | --- | --- | --- | --- | --- | --- |
| **Classes** | **log-likelihood** | **resid. df** | **BIC** | **aBIC** | **cAIC** | **likelihood-ratio** | **Entropy** | **Parameters** | **Smallest class count (n)** | **Smallest class size(%)** |
| Model 1 | -2721.07 | 226 | 5546.67 | 5486.44 | 5565.67 | 2717.91 | - | 19 | 245 | 100 |
| **Model 2** | **-2559.51** | **206** | **5333.57** | **5209.95** | **5372.57** | **2400.14** | **0.767** | **39** | **100** | **41** |
| Model 3 | -2517.41 | 186 | 5359.40 | 5172.37 | 5418.40 | 2317.77 | 0.765 | 59 | 44 | 18 |
| Model 4 | -2483.89 | 166 | 5402.39 | 5151.96 | 5481.39 | 2247.75 | 0.798 | 79 | 54 | 22 |
| Model 5 | -2454.95 | 146 | 5454.53 | 5140.71 | 5553.53 | 2194.80 | 0.777 | 99 | 27 | 11 |
| Model 6 | -2429.79 | 126 | 5514.23 | 5137.01 | 5633.23 | 2140.47 | 0.628 | 119 | 7 | 3 |
