# Supplementary figures and images for: Latent class analysis of the Epidemic‐Pandemic Impacts Inventory on mental health outcomes in Siyan Clinical patients
Source: Health Sci Rep. 2023 Apr 20;6(4):e1215. doi: 10.1002/hsr2.1215 (PMC10119487; doi:10.1002/hsr2.1215)

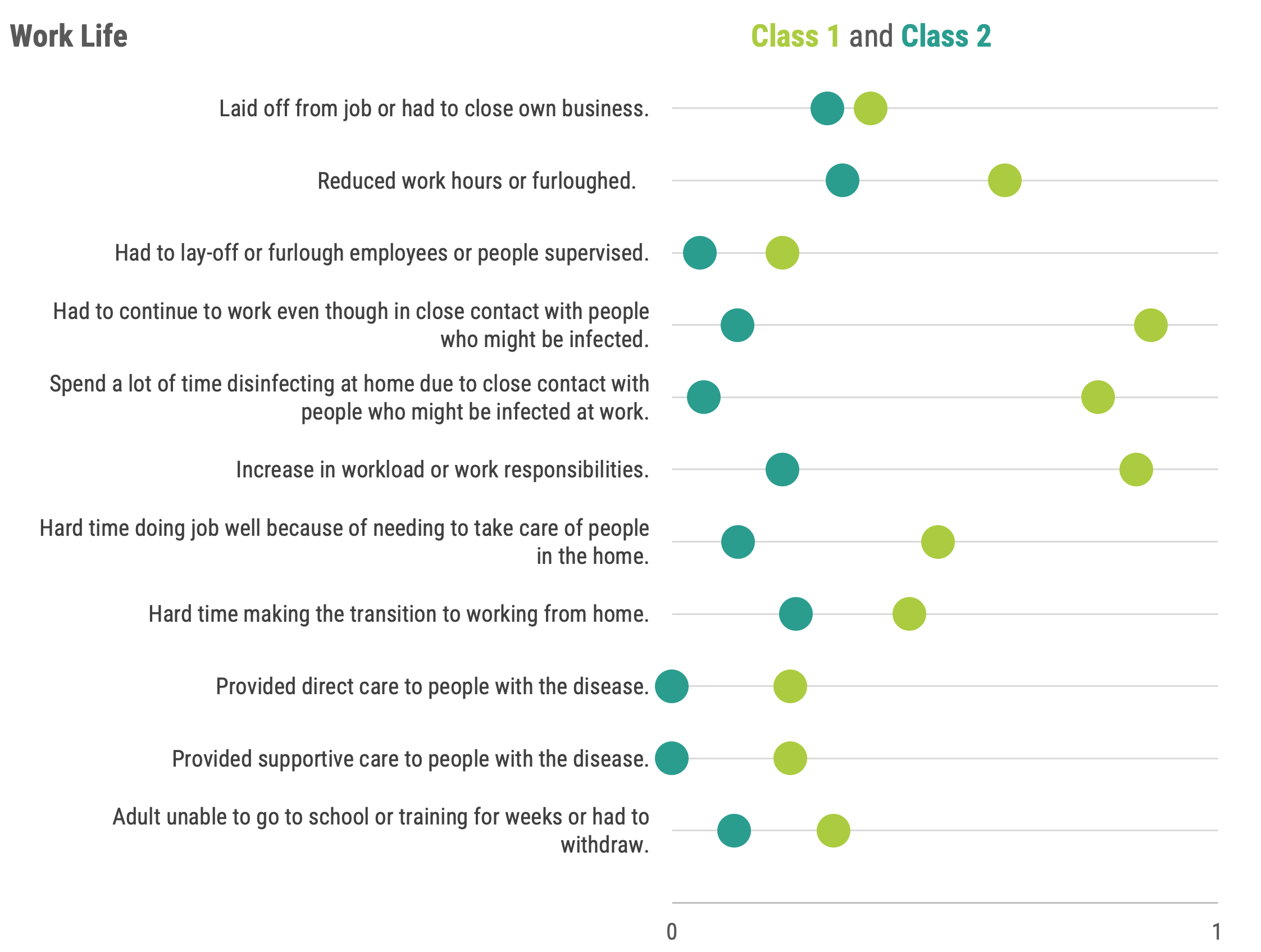

Supplement: Supplementary file 2 — Supplemental Figure 1. [file HSR2-6-e1215-s006.png]

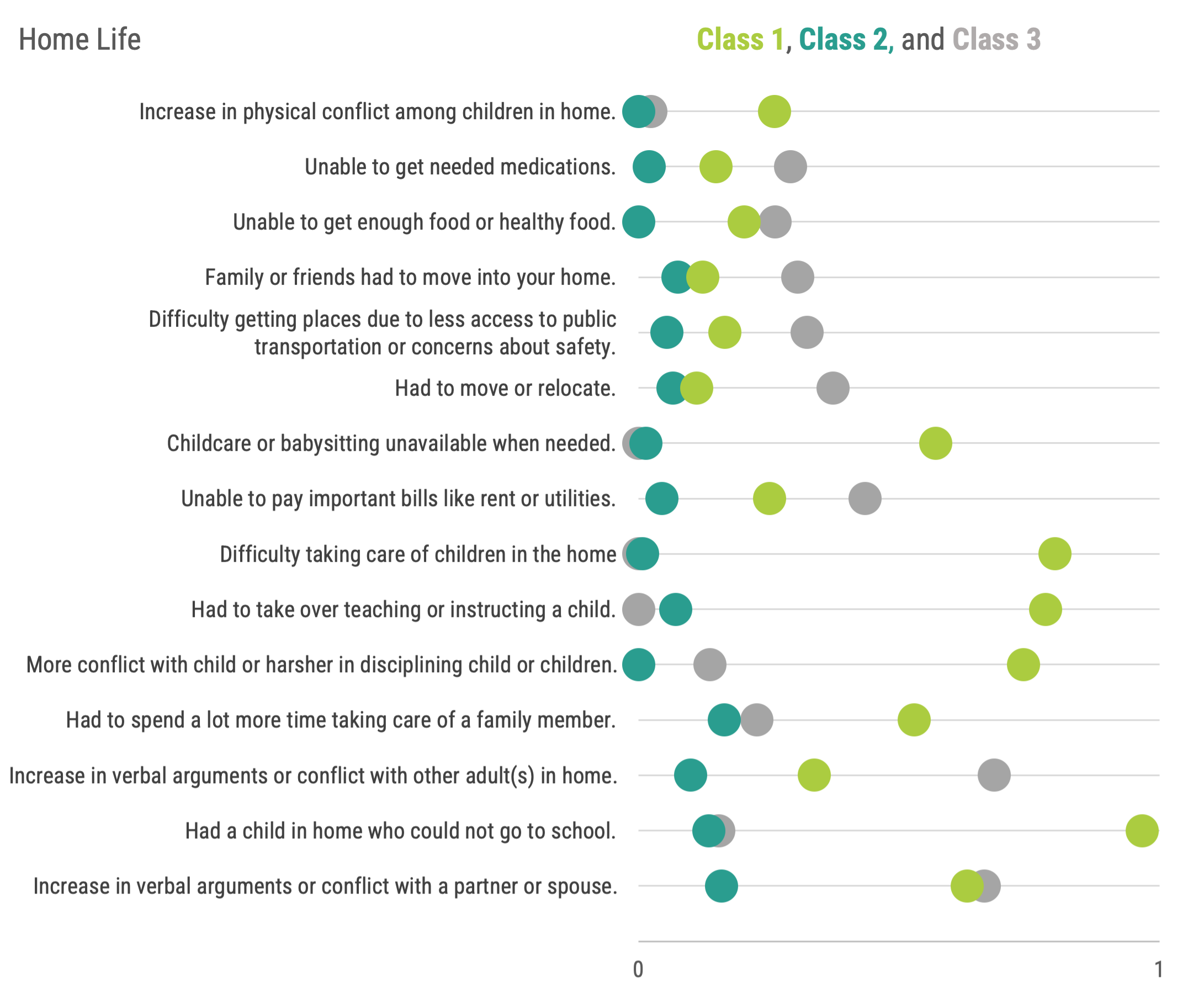

Supplement: Supplementary file 3 — Supplemental Figure 2. [file HSR2-6-e1215-s005.png]

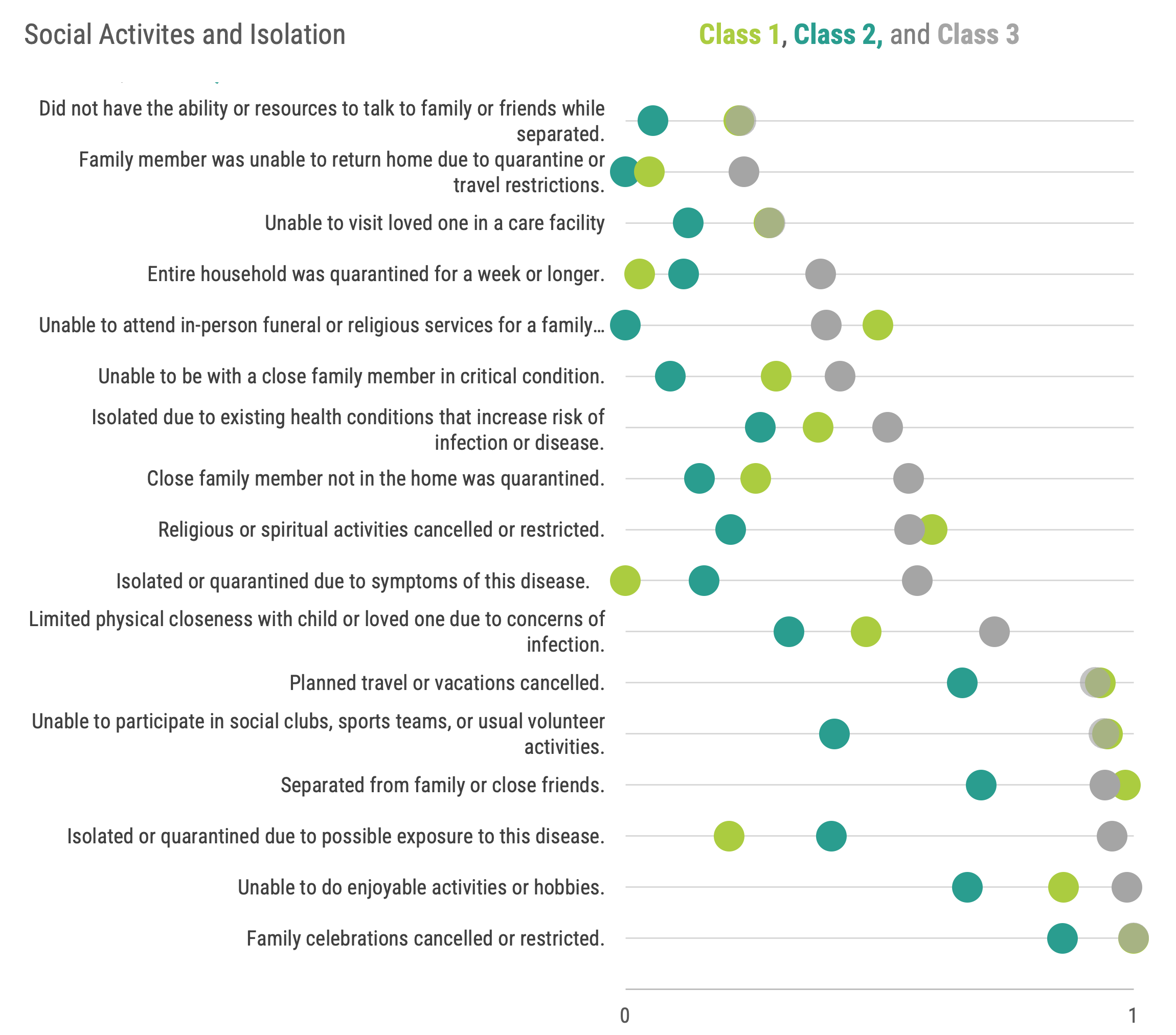

Supplement: Supplementary file 4 — Supplemental Figure 3. [file HSR2-6-e1215-s002.png]

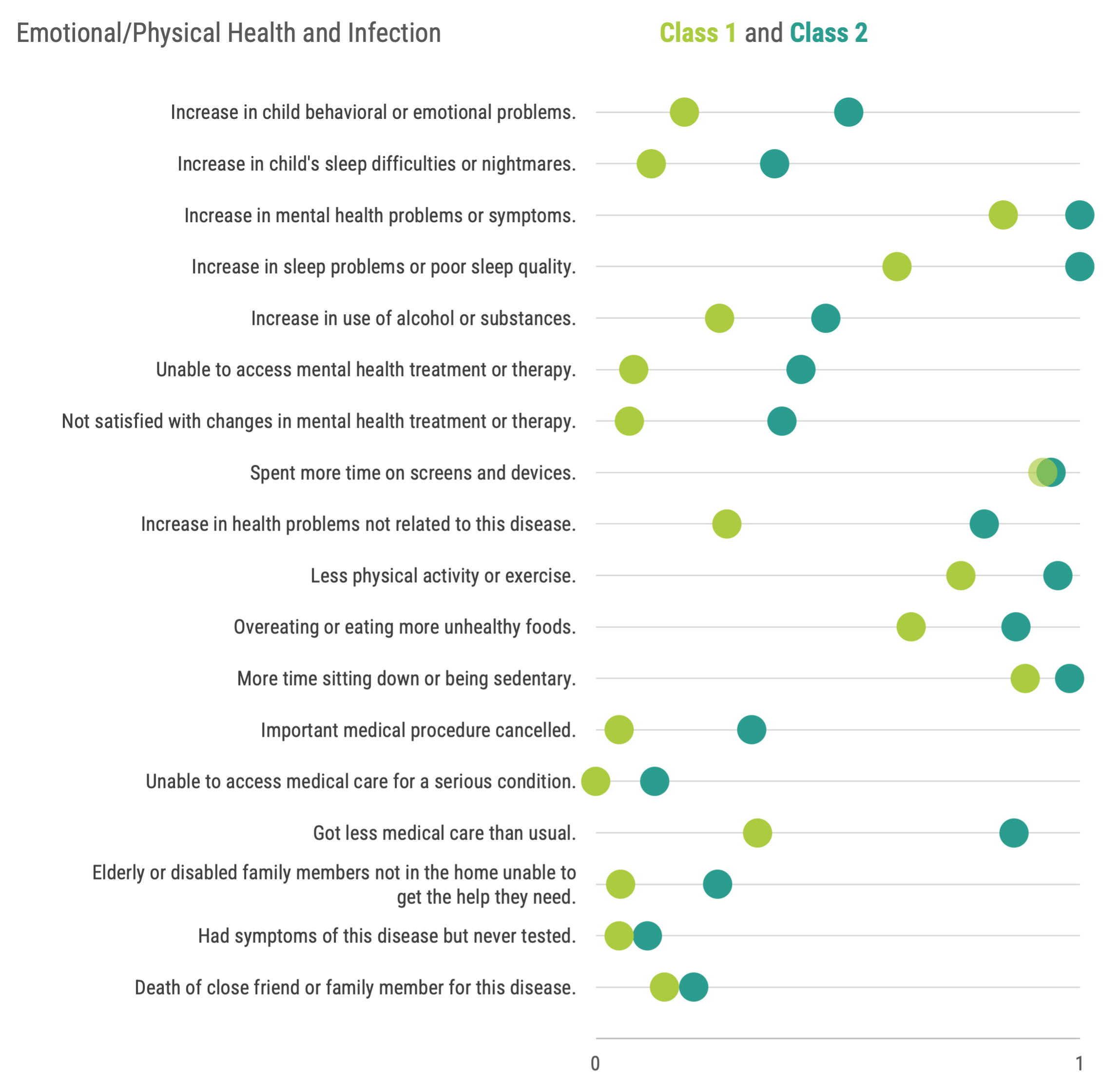

Supplement: Supplementary file 5 — Supplemental Figure 4. [file HSR2-6-e1215-s001.png]

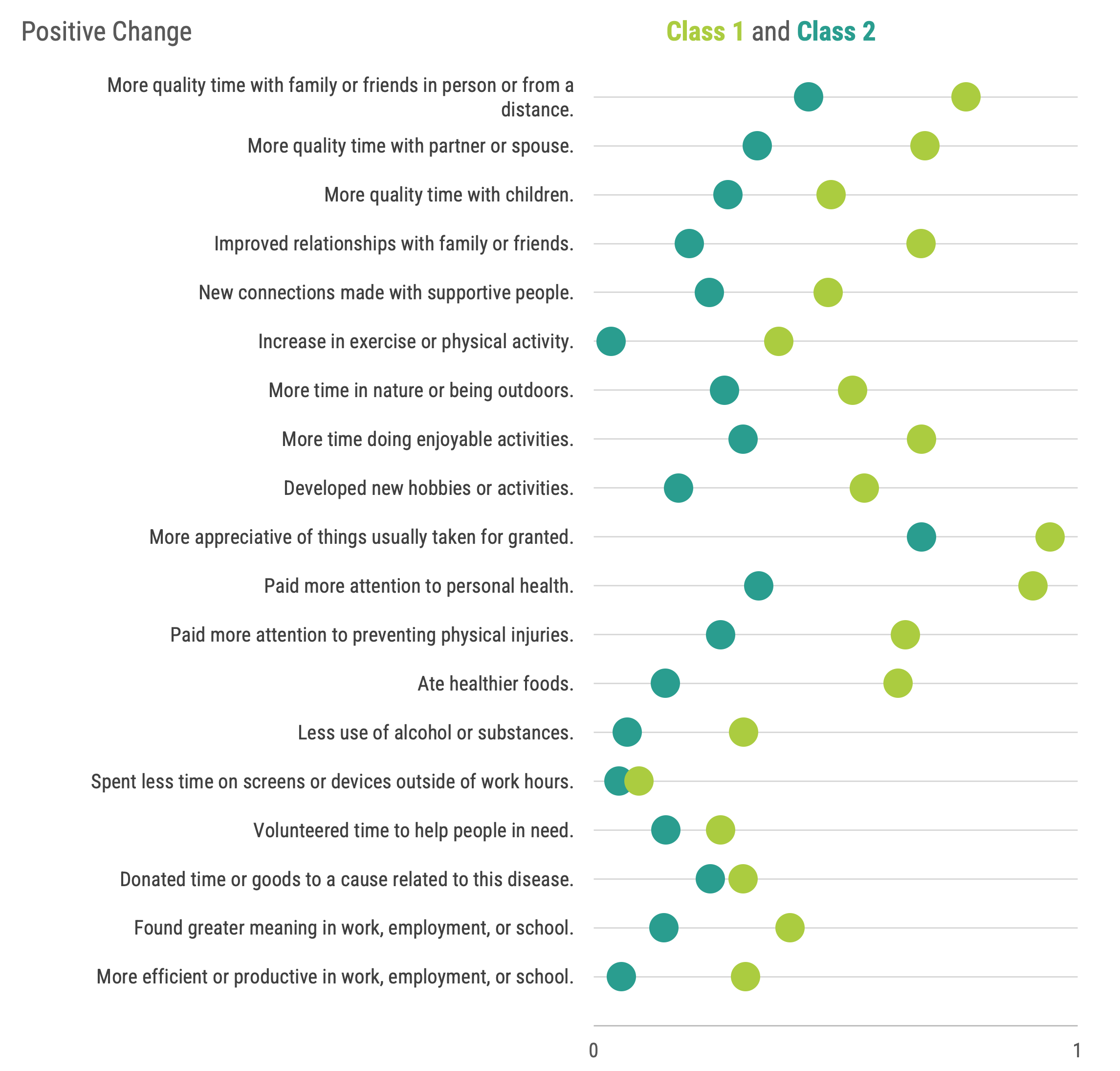

Supplement: Supplementary file 6 — Supplemental Figure 5. [file HSR2-6-e1215-s004.png]
